# Limited efficacy of tocilizumab in adult patients with secondary hemophagocytic lymphohistiocytosis: a retrospective cohort study

**DOI:** 10.1186/s13023-022-02516-1

**Published:** 2022-09-21

**Authors:** Ju Yeon Kim, Miso Kim, Jin Kyun Park, Eun Bong Lee, Jun Won Park, Junshik Hong

**Affiliations:** 1 Division of Rheumatology, Department of Internal Medicine, Seoul National University College of Medicine, 101 Daehak-ro, Jongno-gu, Seoul, 110-744 Republic of Korea; 2 Division of Hematology and Medical Oncology, Department of Internal Medicine, Seoul National University College of Medicine, 101 Daehak-ro, Jongno-gu, Seoul, 110-744 Republic of Korea

**Keywords:** Secondary hemophagocytic lymphohistiocytosis, Interleukin-6, Tocilizumab, HLH-2004 protocol

## Abstract

**Background:**

Interleukin (IL)-6 is one of the key cytokines in the pathogenesis of secondary hemophagocytic lymphohistiocytosis (sHLH); however, the efficacy and safety of tocilizumab (TCZ), a monoclonal IL-6 receptor antibody, in patients with sHLH is uncertain.

**Methods/Results:**

This study included 64 adult patients who were diagnosed with sHLH based on the HLH-2004 criteria. Patients were classified into two groups based on treatment regimen at baseline: tocilizumab (TCZ group, n = 8) *versus* other treatments (control group), including HLH-2004 protocol (n = 35), chemotherapy (n = 7), glucocorticoid alone (n = 8), and with other immunosuppressants (n = 6). Primary outcome was overall 8-week survival. Baseline characteristics between the two groups were comparable. At day 56, one patient (12.5%) in the TCZ group and twenty-eight patients (51.9%) in the control group survived. Univariable and multivariable Cox proportional hazard analysis showed that TCZ significantly increased the risk of death (adjusted hazard ratio 5.55; 95% CI 2.13–14.49). The complete or partial response rate at day 14 was 44.6% in the control group, and nil in the TCZ group. In contrast, infectious complications occurred more frequently in the TCZ group than in the control group (14.3% vs. 50.0%).

**Conclusion:**

Our results suggest that tocilizumab has limited efficacy in treating adult patients with sHLH and could increase the risk of infectious complications compared to the conventional treatments.

**Supplementary Information:**

The online version contains supplementary material available at 10.1186/s13023-022-02516-1.

## Introduction

Hemophagocytic lymphohistiocytosis (HLH) is an aberrant hyper-inflammatory response characterized by excessive cytokine release and abnormal activation of macrophages and T cells, that eventually leads to devastating multi-organ failure [1, 2]. HLH is classified into two subtypes: primary and secondary HLH (sHLH). The former comprises a group of genetic disorders characterized either by loss-of-function mutations in granule-mediated cytotoxicity or by other congenital immunodeficiency syndromes, while the latter is a clinical syndrome that develops in the context of infections, malignancies, autoimmune/auto-inflammatory conditions, or immunotherapy [3, 4].

As untreated HLH is rapidly progressive and life-threatening, prompt initiation of HLH-specific treatment is essential for improved treatment outcomes and prognosis. The HLH-94 and HLH-2004 treatment protocols, which include etoposide, dexamethasone, and cyclosporine, have been established as conventional therapies for pediatric HLH by international, prospective trials [5–7]. In contrast, no standardized treatment strategy has been delineated for HLH in adults on account of its rarity and heterogeneity, which has hampered the successful execution of large scale prospective clinical trials. Therefore, the treatment strategies for HLH in adults, mostly secondary, have been largely extrapolated from those used in pediatric HLH [8, 9]. However, concerns regarding pre-existing comorbidities in elderly patients and treatment-related toxicities such as infectious complications have prompted the search for alternative treatment strategies that target specific immune pathways or cytokine signaling [10–12].

Tocilizumab (TCZ) is a monoclonal antibody directed against the interleukin (IL)-6 receptor. IL-6 has been linked to cytokine release syndrome [13]. TCZ has demonstrated high efficacy and is approved by the U.S. Food and Drug Administration (FDA) for HLH-like cytokine release syndrome after chimeric antigen receptor T-cell (CAR-T) immunotherapy [14, 15]. The syndrome is characterized by marked elevation of IL-6, which is thought to play a key role in the characteristic cytokine storm. In COVID-19 infection, IL-6 levels correlate with disease severity, and anti-IL-6 therapy in critically ill patients demonstrated favorable efficacy in a few randomized trials [16–18]. Furthermore, TCZ was shown to be efficacious in patients with reactive HLH or macrophage activation syndrome (MAS) in a few case reports, suggesting that IL-6 blockade could be a potential therapeutic target in sHLH [11, 19]. The present study aimed to assess the efficacy and safety of IL-6 blockade by TCZ in adult patients with sHLH, as compared to conventional treatments.

## Methods

### Patients

This retrospective study included patients with sHLH who were treated at the Seoul National University Hospital between January 2004 and December 2019. A list of eligible patients was retrieved from the Seoul National University Hospital Patients Research Environment (SUPREME) (https://supreme.snuh.org). This system crosslinks ICD-9 and ICD-10 codes. Using a word “hemophagocytic,” which included patients with the ICD-10 codes of D76.1 and D76.2. Patients with ICD-10 code of D76.3 were additionally reviewed, and none of them had HLH. Fulfillment of at least five of the eight modified HLH-2004 criteria for hemophagocytic lymphohistiocytosis, namely fever, splenomegaly, bicytopenia (hemoglobin < 9 g/dL, platelet count < 100,000/µL, absolute neutrophil count < 1,000/µL), hypertriglyceridemia or hypofibrinogenemia, elevated serum ferritin > 3,000 ug/L, elevated soluble IL-2 receptor > 2,400 units/mL, low or absent natural killer cell cytotoxicity, and tissue demonstration of hemophagocytosis led to inclusion in the study [6, 10, 20]. All patients younger than 18 years of age, those without available medical records, and those that were diagnosed with pHLH were excluded.

The study was approved by the Seoul National University Hospital Institutional Review Board (IRB approval number: 2105-107-1219), and all research was performed in accordance with relevant guidelines/regulations. Informed consent was waived by the IRB due to the retrospective nature of the study.

### Clinical data

All medical records of eligible patients regarding their demographic details, medication history, laboratory and radiographic investigations, adverse events, and the date and cause of death were collected. The H-score was calculated based on a set of weighted criteria as described by Fardet et al. [21]. The baseline date was defined as the date when a specific treatment for sHLH (index treatment) was initiated. Included patients were classified into two groups based on the treatment regimen initiated at baseline, which was either TCZ treatment or all other treatments including the HLH-2004 protocol, other chemotherapeutic agents, and glucocorticoids with or without other immune modulating agents (*i.e.*, TCZ group vs. control group).

### Response assessment

Treatment response was assessed by evaluation of all medical records of clinical and laboratory findings that were maintained daily until the date of discharge and weekly thereafter until week 8. Treatment response was assessed based on the following clinical parameters: 1) complete blood count profile, 2) coagulopathy, 3) neurologic manifestations, 4) hepatitis and/or hyperbilirubinemia, 5) hemodynamic instability, 6) renal insufficiency, 7) hepatosplenomegaly and 8) fever. A complete response (CR) was defined as the normalization of all abnormal clinical and laboratory findings. An improvement of at least 25% in two or more abnormal clinical or laboratory findings was defined as a partial response (PR), while a worsening of more than 50% in two or more of the same was categorized as progressive disease (PD). Patients who fulfilled neither of these criteria were classified as having stable disease (SD) [10]. The primary outcome of the study was overall eight-week survival. Secondary outcomes included the proportion of patients who achieved an overall response (including CR and PR) and the proportion of patients who experienced the composite outcome (PD and death) at D14, D28, and D56. In addition, the occurrence of infectious complications was assessed throughout the observation period.

### Statistical analysis

We performed an intention-to-treat analysis for all efficacy and safety assessments. Continuous or dichotomous baseline data were compared using the Wilcoxon rank sum test or Fisher’s exact test, as appropriate. Overall survival between the two groups was compared using the log-rank test and the Cox proportional hazard model. Efficacy outcomes between the two groups were analyzed using a binary logistic regression analysis. In the multivariable analysis, clinical factors with relevant associations (*P* < 0.1) with the outcome in the univariate analysis were included as covariates. Firth’s penalized maximum likelihood was used when the outcome variables showed a complete separation.

All statistical analyses were performed using IBM SPSS version 26 (IBM Corp., Armonk, NY, USA). Statistical significance was set at *P* < 0.05.

## Results

### Baseline characteristics

A total of 77 patients with sHLH who fulfilled the HLH-2004 diagnostic criteria were included in the study. Among them, 13 patients were excluded because they experienced rapid disease progression and early death before the initiation of any treatment for sHLH. The remaining 64 patients who received at least one HLH-targeted treatment were analyzed. Of these, eight patients received TCZ as the first-line therapy (TCZ group)—four patients received doses of 4 mg/kg, two received 8 mg/kg, and one received 6 mg/kg. Only two of these patients survived long enough to receive a second dose of TCZ two weeks following the initial dose; the rest only received TCZ once (Additional file 1: Table S1). Fifty-six patients received treatments other than TCZ (control group), which consisted of HLH-2004 regimen (n = 35), malignancy targeted chemotherapy (n = 7), glucocorticoid alone (n = 8), and other immunosuppressive therapies (n = 6). Four of whom initially treated with HLH-2004 protocol subsequently received cancer-specific chemotherapy. None of the patients in the control group received Janus kinase (JAK) inhibitors, such as ruxolitinib, at baseline. The inclusion and exclusion criteria as well as the list of individual treatments given to the controls are presented in Additional file 1: Fig. S1.

Baseline characteristics are listed in Table 1. The etiologies of sHLH were comparable, but the control group had a greater number of patients with malignancies. All cases were experiencing the first episode of sHLH except for one case in the TCZ group, in which disease had been reactivated after a period of remission. The patients who were diagnosed with malignancy associated HLH initiated baseline treatment earlier (mean 29.2 ± 15.7 days vs. mean 45.3 ± 31.0 days, *P* = 0.048).

**Table 1:** Baseline characteristics of the patients

	Control group (n = 56)	TCZ group (n = 8)	*P*
Age, years, n	47.3 (17.5)	48.5 (19.7)	0.871
Sex, male, n	24 (42.9)	4 (50.0)	0.721
Cause of HLH			
Rheumatic disease	9 (16.1)	3 (37.5)	0.164
Malignancy	17 (30.4)	1 (12.5)	0.424
Infection	13 (23.2)	2 (25.0)	1.000
Idiopathic	18 (32.1)	1 (12.5)	0.418
ICI	0 (0)	1 (12.5)	0.125
Symptom-to-treatment interval, days	41.0 (29.4)	33.9 (15.0)	0.730
Prior chemotherapy^a^	1 (1.8)	1 (12.5)	0.236
Prior immunosuppressant^a^	4 (7.1)	2 (25.0)	0.159
Glucocorticoid before baseline, n	37 (66.1)	8 (100)	0.093
Baseline Glucocorticoid dose, mg/day^b^	112.9 (49.0)	113.3 (21.4)	0.396
Baseline clinical factors			
Fever	54 (96.4)	8 (100.0)	1.000
Hepatosplenomegaly	50 (89.3)	8 (100.0)	1,000
CNS manifestation^c^	15 (26.8)	3 (37.5)	0.676
Hepatitis	49 (87.5)	6 (75.0)	0.312
Shock	28 (50.0)	4 (50.0)	1.000
Azotemia	11 (19.6)	2 (25.0)	0.660
Coagulopathy	47 (83.9)	8 (100.0)	0.587
H-score	243.3 (36.8)	233.6 (20.1)	0.422
GFR, mL/min/1.73 m^2^	108 (77.3)	106.1 (75.5)	0.984
MELD score	12.5 (7.2)	12.2 (9.2)	0.509
ANC, /uL	2619.8 (5898.6)	4691.5 (5472.0)	0.345
Hemoglobin, g/dL	8.6 (2.1)	9.7 (3.0)	0.477
Platelet, x10^3^/μL	50.5 (34.4)	59.0 (40.7)	0.670
Ferritin, μg/L	27,827.6 (55,539.4)	20,873.7 (15,333.7)	0.283
Fibrinogen, mg/dL	161.4 (125.3)	155.1 (90.6)	0.879

Data are presented as the mean (SD) or n (%)

ANC, absolute neutrophil count; CNS, central nervous system; GFR, glomerular filtration rate; HLH, hemophagocytic lymphohistiocytosis; ICI, immune checkpoint inhibitor; MELD, Model For End-Stage Liver Disease; TCZ, tocilizumab

^a^Defined as any prior history of chemotherapy or immunosuppressant prescription before the baseline treatment

^b^Presented as prednisolone equivalent dose

^c^An altered level of consciousness

There were no significant differences in the clinical features at baseline between the two groups. The median interquartile range (IQR) and *H*-score was 247.0 and 64 respectively for the control group and 237.0 and 33 respectively for the TCZ group, both of which indicated a greater than 98% probability of HLH [21]. Azotemia, defined as estimated glomerular filtration rate (eGFR) < 60, was seen in 19.6% (11/56) of the control group, and 25% (2/8) of the TCZ group, and both groups had a mean Model for End-Stage Liver Disease (MELD) score of greater than 11 at baseline. The mean (SD) time interval from symptom onset to baseline in the control and TCZ group were 41.0 (29.4) and 33.9 (15.0) days, respectively, which were not significantly different (*P* = 0.730). Prior to sHLH targeting therapy at baseline, all eight patients (100.0%) in the TCZ group and 37 of the 56 (66.1%) patients in the control group were receiving glucocorticoid. After initiating the index treatment, TCZ or control therapies, all patients in both groups concomitantly started high dose glucocorticoid, at a dose of at least 1 mg/kg prednisolone equivalent dose.

### Treatment responses

**Fig. 1:**
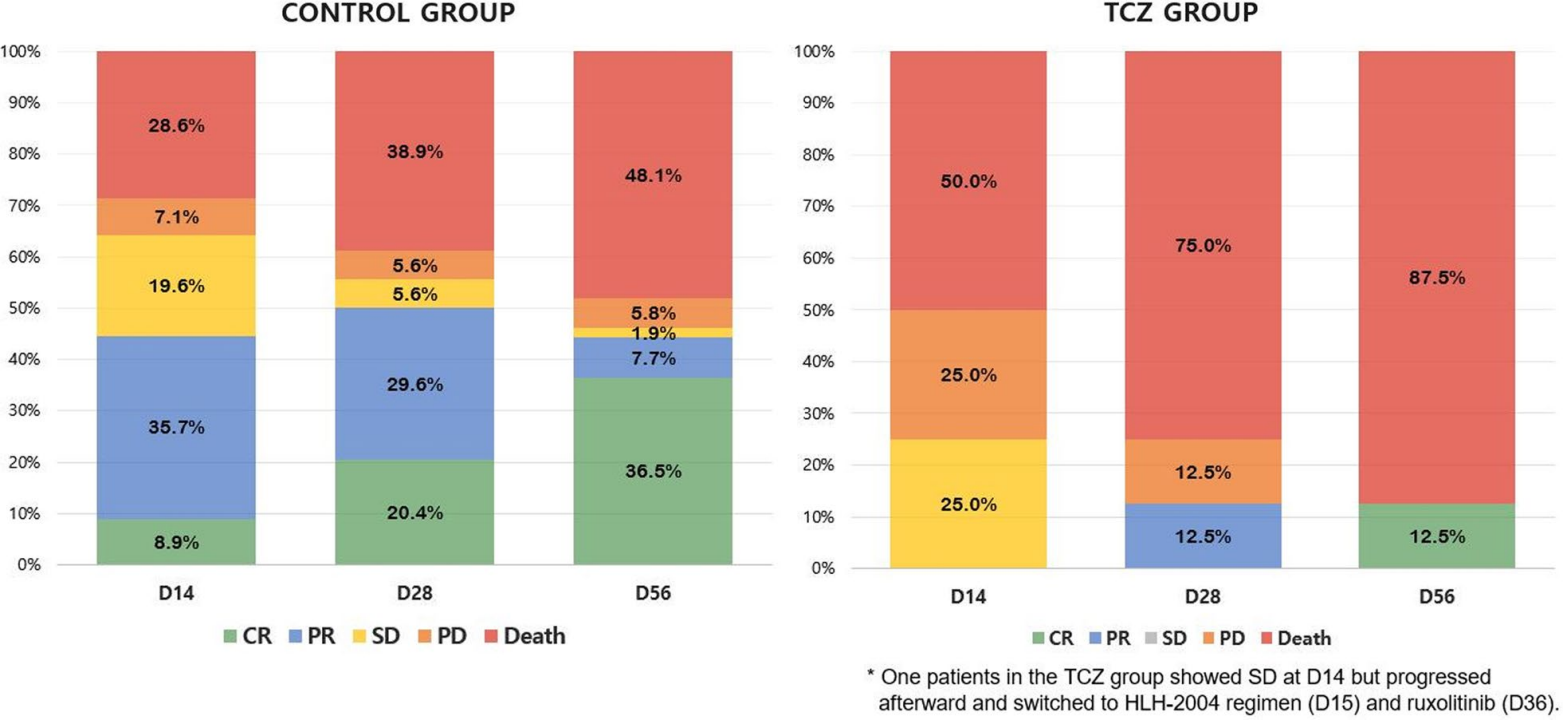
Treatment response at D14, D28, and D56 was assessed as complete response (CR), partial response (PR), stable disease (SD), progressive disease (PD), or death in the two groups

At eight weeks from baseline, 12.5% (1/8) of the patients in the TCZ group and 51.9% (27/52) of the patients in the control group had survived. All deaths were attributable to the progression of sHLH and/or its associated complications, which were mostly infections. The cause of death and the duration of survival of the patients in the TCZ group are presented in Additional file 1: Table S1. Univariable Cox proportional hazard analysis revealed that TCZ was significantly associated with an increased risk of death at D56 (hazard ratio [HR] 2.92; 95% CI 1.25–6.82) (Table 2). This result was consistent with that observed in the multivariable analysis, in which relevant clinical factors were included as covariates (adjusted HR = 5.55; 95% CI 2.13–14.49) (Fig. 1, Table 2). Regarding the underlying etiologies, rheumatic disease, malignancy, and infection were not significantly associated with an increased risk of death at D56.

**Table 2:** Clinical factors associated with 8-week overall survival

	Univariable analysis		Multivariable analysis^*^	
	HR (95% CI)	*p*	HR (95% CI)	*p*
Clinical factors				
Age^†^	1.03 (1.00–1.05)	**0.020**	1.00 (0.98–1.03)	0.714
Male sex	0.68 (0.34–1.36)	0.272		
Symptom duration, days^†^	1.00 (0.99–1.01)	0.450		
Steroid use prior to the treatment	0.84 (0.39–1.81)	0.653		
Baseline MELD^†^	1.09 (1.04–1.14)	**< 0.001**	1.09 (1.03–1.16)	**0.005**
Baseline GFR, mL/min/1.73 m^2†^	0.99 (0.99–1.00)	**0.064**	1.00 (1.00–1.01)	0.623
Baseline H-score^†^	1.00 (0.99–1.01)	0.524		
Baseline fibrinogen, mg/dL^†^	1.00 (0.99–1.00)	0.144		
Underlying cause				
Rheumatic disease	0.93 (0.38–2.25)	0.864		
Malignancy	0.49 (0.20–1.20)	0.119		
Infection	0.82 (0.35–1.88)	0.632		
Idiopathic	2.21 (1.09–4.49)	**0.028**	2.44 (1.11–5.36)	**0.027**
Treatment at baseline				
HLH-2004 regimen	0.69 (0.35–1.39)	0.302		
Chemotherapy	0.36 (0.09–1.52)	0.166		
Other immunosuppressant	0.54 (0.13–2.26)	0.400		
Steroid only	3.21 (1.30–7.95)	**0.012**	2.85 (0.98–8.30)	0.056
Tocilizumab	2.92 (1.25–6.82)	**0.013**	5.55 (2.13–14.49)	**< 0.001**

^Bold text indicates a statistically significant difference with a p-value less than 0.05^

^*^Adjusted for clinical factors with relevant association (*P* < 0.1) in univariable analysis

^†^The variable was included in the model as a continuous variable

GFR, glomerular filtration rate; HLH, hemophagocytic lymphohistiocytosis; MELD, Model For End-Stage Liver Disease

**Fig. 2:**
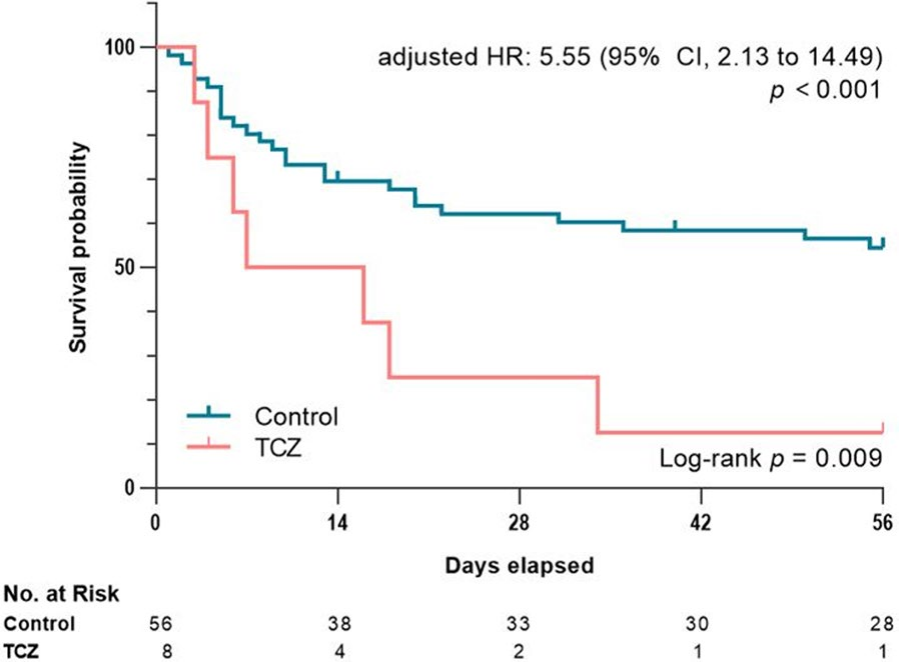
Kaplan–Meier curve for eight-week survival in the two groups. TCZ-tocilizumab group

The overall response rates in the control group at D14, D28, and D56 were 44.6% (25/56), 50.0% (27/54), and 44.2% (23/52), respectively. A complete response was achieved in 8.9% (5/56) in D14, 20.4% (11/54) in D28, 36.5% (19/52) in D56; a partial response was observed in 35.7% (20/56), 29.6% (16/54), and 7.7% (4/52), respectively. In contrast, no patient showed a partial or complete response at D14 in the TCZ group (Fig. 2). Only one patient, who switched from TCZ to the HLH-2004 regimen at D15 and to ruxolitinib at D35 due to limited response to treatment, had a favorable response at D28 (PR) and D56 (CR) in the TCZ group.

By D56, composite outcome had occurred in 87.5% (7/8) of the patients in the TCZ group and 53.9% (28/52) of the patients in the control group (Fig. 2). The univariable and multivariable logistic regression analyses revealed that TCZ treatment significantly increased the risk of composite outcome at D56 (adjusted OR 12.93; 95% CI 1.16–144.05) (Additional file 1: Table S2). In addition, the TCZ group was also significantly associated with more frequent composite outcomes at D14 (Additional file 1: Table S3) and D28 (Additional file 1: Table S4).

### Infectious complication

A total of 24 patients experienced at least one infectious complication during the observation period. The most common infection was bacteremia (n = 12), followed by pneumonia/lower respiratory tract infections (n = 11) and fungemia (n = 8) (Table 3). The proportion of patients who experienced at least one infectious complication was higher in the TCZ group (62.5%) as compared to the control group (33.9%), with a P value of 0.139. The patients who had malignancy associated HLH had a lower number of infections during the treatment course (16.7% (3/18) vs 45.7% (21/46), *P* = 0.031). At the time of infection, 14 of 19 (73.7%) in TCZ group and 3 of 5 (60%) in control group, were neutropenic (*P* = 0.608). As many as 14 patients were infected with multiple strains of microorganisms simultaneously, and the proportion of such patients was higher in the TCZ group than in the control group (50% vs. 17.9%, *P* = 0.062). The proportion of patients who were detected with bacteremia were significantly higher in the TCZ group.

**Table 3:** Infectious complication during the observation period

	Control group (n = 56)	TCZ group (n = 8)	*P*
At least one infectious complication	19 (33.9)	5 (62.5)	0.139
Bacteremia	8 (14.3)	4 (50.0)	0.035
Fungemia	5 (8.9)	3 (37.5)	0.054
Pneumonia/lower-respiratory tract infection	10 (17.9)	1 (12.5)	1.000
GI infection	4 (7.1)	0 (0.0)	1.000
Other infections	3 (5.4)	0 (0.0)	1.000
Infection of unknown focus	1 (1.8)	0 (0.0)	1.000

GI, Gastrointestinal; TCZ, tocilizumab

There were standardized anti-microbial prophylaxis for HLH-2004 protocol and high dose glucocorticoid. There was no standardized anti-microbial prophylaxis protocol for TCZ infusion. Nevertheless, of the 8 patients who received TCZ, 6 were receiving broad spectrum empirical antibiotics and 5 were receiving antifungal agents, because these patients were experiencing recurrent fever and/or neutropenia.

### Sensitivity analysis

At the first instance, we only included patients who were treated with the HLH-2004 regimen in the control group and compared the eight-week overall survival between this group and the TCZ group. Both univariate and multivariate analyses showed that the TCZ group was significantly associated with a poor prognosis (Additional file 1: Table S5 and Fig. S2). Next, we compared the overall survival, after defining the baseline date as the date of symptom onset to account for the possible confounding effect of delayed treatment. The results thus obtained were consistent with those seen in the original Cox regression analysis (Additional file 1: Table S6 and Fig. S3). Lastly, we evaluated the HLH response based on a set of response criteria described in the emapalumab study [22]. As consistent with our study, the TCZ group had a lower response rate than the control group, although not statistically significant. At D56, 42.9% (24/56) and 12.5% (1/8) of control and TCZ group respectively had overall response, which consist of CR, PR, and improvement (adjusted OR 0.11 [0.01 to 1.30], *P* = 0.113) (Additional file 1: Fig. S4).

## Discussion

To the best of our knowledge, this is the first study that investigates the safety and efficacy of IL-6R inhibition in comparison to conventional treatments in patients with sHLH. A higher proportion of patients in the TCZ group experienced disease progression and showed a poor survival rate as compared to those in the control group. Furthermore, infectious adverse events occurred more frequently in the TCZ group. These results suggest that the safety and efficacy of TCZ is not favorable in patients with sHLH as compared to conventional treatment options.

As hypercytokinemia is a pathological hallmark of HLH, and consequently cytokine-targeted therapies are of particular interest in this era of biologic therapies, the results of this study are significant. However, selective neutralization of a single cytokine within the panoply of cytokines engaged in HLH may not always be effective in terminating disease progression. Tumor necrosis factor-α, interferon-γ, and various interleukins, including IL-1, IL-2, IL-6, and IL-18, have been linked to the cytokine cascade implicated in sHLH, and each of these cytokines has a complex interconnection with each other, that is not fully understood [23, 24]. Among the cytokine milieu, IL-6 is a part of the downstream cascade induced by IL-1β and IL-18, rather than a driver cytokine. Therefore, it is uncertain if treatment with TCZ alone has a significant effect on the various inflammatory cytokines involved in sHLH. Moreover, despite the typical IL-6 elevation in the preclinical models of sHLH and other cytokine release syndromes, the role of IL-6 in the pathogenesis of HLH remains unclear. A previous study on the cytokine pattern in HLH showed that IL-6 is only modestly elevated in HLH, contrary to what was observed for other key cytokines [25]. It is of note that two dermatomyositis patients in the TCZ group also had a poor treatment outcome. Previous studies suggested that dermatomyositis-related MAS is associated with high mortality rate but no standard treatment strategy has been established yet [26, 27]. Although a few studies reported cases of anti-MDA-5 amyopathic dermatomyositis complicated by MAS that were successfully treated with TCZ, these patents were concomitantly treated with glucocorticoid and other immunosuppressants, such as cyclosporine and rituximab [28]. Considering that excessive type I interferon activity, of which most up-regulated in patients with dermatomyositis, has also been implicated in a HLH-like syndrome, targeting IL-6 alone may not be sufficient in controlling disease activity [29, 30]. In light of these findings, clinicians should be precautious when using TCZ as a monotherapy in patients with dermatomyositis-related MAS.

As all the patients in the TCZ group were treated with TCZ as the frontline therapy, the poor prognosis in this group is attributable to the limited efficacy of TCZ, rather than to the selection of refractory cases in the TCZ group. Our result is contrary to that reported in a recent case series, which showed clinical remission in eight of nine sHLH patients treated with TCZ [11]. However, the time to reach remission, response duration, and time interval to death were not clearly described in the same study. Furthermore, four (50.0%) of the patients who achieved remission eventually died during hospitalization, and another three patients developed septic shock. This finding is in line with our result that 62.5% of the patients in the TCZ group experienced at least one infectious complication. Similar trends have been reported in other studies [31, 32]. Lang et al. reported that patients treated with TCZ showed higher rates of infectious complication [31]. Furthermore, a recent study on patients with COVID-19 related cytokine release syndrome demonstrated that late-onset infections (> 48 h following admission) were more common among those who received TCZ [26]. Although the cause of this phenomenon is undeciphered, discrepancies in the levels of acute phase reactants such as C-Reactive Protein following the administration of TCZ, which might mask the typical signs of infection and therefore delay diagnosis have been previously reported [32, 33].

Contrary to previous reports, overall survival in patients with malignancy-associated HLH was not inferior to that in those with other underlying diseases [34–36]. It could be attributable to the fact that patients with non-malignancy associated HLH were diagnosed at a later stage of the disease. This delay in diagnosis could lead to delay in treatment and consequentially more complications before the treatment. In fact, the patients who were diagnosed with malignancy associated HLH initiated baseline treatment earlier and had a lower number of infections during the treatment course. A similar result was observed in a case series performed in Latin America [37].

This present study has several limitations. First, on account of the study not being a randomized study, its results could be biased by “confounding by indication.” Although we have demonstrated that the baseline characteristics such as duration of symptoms, clinical manifestations, and H-score were comparable between the two groups, imbalances of unmeasured factors such as preferences of the treating physicians could have influenced the outcome. Second, the number of patients in the TCZ group was relatively small, which was not unexpected given the rarity of the disease. A small sample size could lead to an overestimation of effect size and low reproducibility. Although we repeatedly found inferior outcome associated with TCZ treatment in sHLH after performing several sensitivity analyses, the results should be consistently replicated and verified in subsequent studies before being put into real practice. Third, the patients in TCZ group were more recently treated when compared to the control. However, this time difference did not shift the bias toward the positive outcome of TCZ. Finally, the sample size of this study was too small to perform a meaningful subgroup analysis that can be stratified based on etiology or specific individual treatment regimen.

## Conclusion

In summary, our results suggest that TCZ treatment has limited efficacy and is associated with an increased risk of infectious complications as compared to conventional treatment in patients with sHLH. Although the safety and efficacy of TCZ should be further investigated and clarified by randomized studies in the future, its use should be reconsidered until robust evidence supporting its application for treatment of sHLH emerges.

## Supplementary Information

**Additional file 1**. Supplement Material: supplemntary figure S1 to S3 and supplementary table S1 to S6. **Supplementary figure S1.** Flow of patient inclusion. **Supplementary table S1.** Summary of patients in the tocilizumab group. **Supplementary table S2.** Risk factor for composite outcome at D56. **Supplementary table S3**. Risk factor for composite outcome at D14. **Supplementary table S4.** Risk factor for composite outcome at D28. **Supplementary table S5.** Clinical factors significantly associated with 56-day overall survival in the subgroup of patients who were treated with tocilizumab or HLH-2004 regimen. **Supplementary figure 2.** Kaplan-Meier curve comparing Day 56 overall survival in the subgroup of patients : tocilizumab versus HLH-2004 regimen. **Supplementary table S6.** Clinical factors significantly associated with overall survival from the onset of initial HLH-related symptom. **Supplementary figure 3.** Kaplan-Meier curve between the two groups from the onset of initial HLH-related symptom.

## Data Availability

The datasets used and/or analysed during the current study available from the corresponding author on reasonable request.

## Abbreviations

CR: Complete response
HR: Hazard ratio
HLH: Hemophagocytic lymphohistiocytosis
IRB: International review board
IL: Interleukin
PR: Partial response
pHLH: Primary hemophagocytic lymphohistiocytosis
PD: Progressive disease
sHLH: Secondary hemophagocytic lymphohistiocytosis
SD: Stable disease
TCZ: Tocilizumab
