# Supplementary material for: Limited efficacy of tocilizumab in adult patients with secondary hemophagocytic lymphohistiocytosis: a retrospective cohort study
Source: Orphanet J Rare Dis. 2022 Sep 21;17:363. doi: 10.1186/s13023-022-02516-1 (PMC9490693; doi:10.1186/s13023-022-02516-1)
Supplement: Supplementary file 1 — Additional file 1. Supplement Material: supplemntary figure S1 to S3 and supplementary table S1 to S6. Supplementary figure S1. Flow of patient inclusion. Supplementary table S1. Summary of patients in the tocilizumab group. Supplementary table S2. Risk factor for composite outcome at D56. Supplementary table S3. Risk factor for composite outcome at D14. Supplementary table S4. Risk factor for composite outcome at D28. Supplementary table S5. Clinical factors significantly associated with 56-day overall survival in the subgroup of patients who were treated with tocilizumab or HLH-2004 regimen. Supplementary figure 2. Kaplan-Meier curve comparing Day 56 overall survival in the subgroup of patients : tocilizumab versus HLH-2004 regimen. Supplementary table S6. Clinical factors significantly associated with overall survival from the onset of initial HLH-related symptom. Supplementary figure 3. Kaplan-Meier curve between the two groups from the onset of initial HLH-related symptom. [file 13023_2022_2516_MOESM1_ESM.docx]

**SUPPLEMENTARY MATERIAL**

**Supplementary figure S1. Flow of patient inclusion**


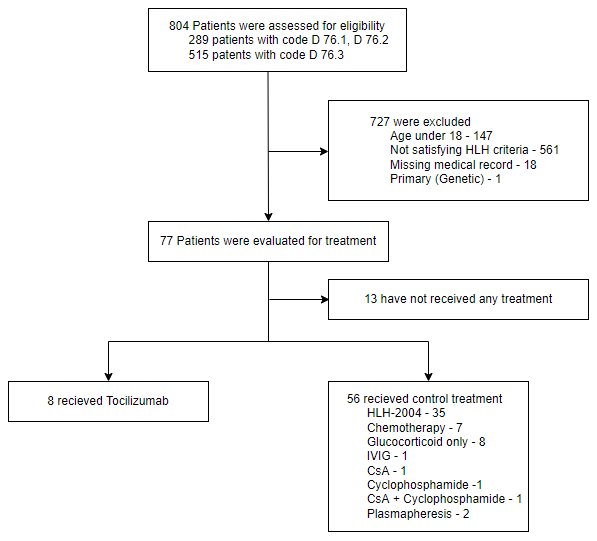


**Supplementary table S1. Summary of patients in the tocilizumab group**

| **No** | **Age** | **Sex** | **Cause of HLH** | **Underlying immunodeficiency** | **TCZ dose,**  **times** | **Other immunosuppressive treatment** | **H-score/**  **ferritin** | **Rescue regimen (Days after TCZ)** | **Outcome at D14** | **Outcome at D28** | **Outcome at D56** | **Cause of death** |
| --- | --- | --- | --- | --- | --- | --- | --- | --- | --- | --- | --- | --- |
| **1** | 52.8 | M | Anetumab ravtansine | Thymic carcinoma | 4 mg/kg, once | mPD 80 mg | 208/  40000 |  | Death (D3) |  |  | MOF due to HLH aggravation |
| **2** | 31.7 | F | Dermatomyositis |  | 8 mg/kg, twice | mPd 1g (D-1 ~ D0) mPD 60 mg | 206/  9103 |  | Death (D4) |  |  | MOF due to HLH aggravation |
| **3** | 65.2 | M | Infection | On Pd 50 mg (Tbc pericarditis) R/O IgG4RD | 4 mg/kg, once | IVIG 0.5g/kg (D-5, D5), mPd 1g (D-3 ~ D0), Dexamethasone 16 mg/day | 226/  45230 |  | Death (D6) |  |  | Candidemia |
| **4** | 62.6 | F | DLBCL | **R-CHOP (D-1) IT-Triple (D-2)** | 4 mg/kg, once | mPD 80 mg | 245/  21920 |  | Death (D7) |  |  | Candidemia |
| **5** | 68.9 | F | Tuberculosis |  | 8 mg/kg, once | Dexamethasone 20 mg/day | 245/  6925 | HLH-2004 (D5) | PD | Death (D16) |  | Septic shock |
| **6** | 23.7 | M | APS |  | 4 mg/kg, once | CsA, PD, Dexamethasone 20 mg/day | 265/  17295 |  | PD | Death (D18) |  | MOF due to HLH aggravation |
| **7** | 21.6 | F | Dermatomyositis | mPD 750-500-500 mg (D-32~-30) IVIG 0.4g/kg/d  (D-32~-29, -18~-14), Cyclophosphamide (D-13) | 8 mg/kg, twice | Dexamethasone 20 mg/day | 244/  2680 |  | SD | Death (D32) |  | MOF due to HLH aggravation |
| **8** | 61.7 | M | ICI | RCC | 6 mg/kg, once | Dexamethasone 20 mg/day | 230/  23837 | HLH-2004 (D14) Ruxolitinb (D35) | SD | PR | CR |  |

APS, anti-phospholipid antibody syndrome; CR, complete response; CsA, cyclosporine A; DLBCL, diffuse large B-cell lymphoma; HLH, hemophagocytic lymphohistiocytosis; ICI, immune checkpoint inhibitor; IVIG, intravenous immunoglobulin; MOF, multiorgan failure; mPd, methylprednisolone; PD, progressive disease; Pd, prednisolone; PR, partial response; RCC, renal cell carcinoma; SD, stable disease.

**Supplementary table S2. Risk factor for composite outcome at D56**

|  | **Univariable analysis** | | **Multivariable analysis^*^** | |
| --- | --- | --- | --- | --- |
|  | **OR (95% CI)** | **P** | **OR (95% CI)** | **P** |
| **Clinical factors** |  |  |  |  |
| **Age** | 1.03 (1.00 to 1.06) | 0.087 | 1.02 (0.99 o 1.06) | 0.255 |
| **Sex, male** | 0.38 (0.14 to 1.06) | 0.065 |  |  |
| **Symptom duration** | 1.03 (1.00 to 1.05) | 0.032 | 1.03 (1.00 to 1.06) | 0.069 |
| **Steroid use prior to the treatment** | 0.80 (0.26 to 2.45) | 0.690 |  |  |
| **Baseline MELD** | 1.16 (1.05 to 1.27) | 0.003 | 1.10 (0.97 to 1.25) | 0.144 |
| **Baseline GFR** | 0.99 (0.98 to 1.00) | 0.050 | 0.99 (0.98 to 1.00) | 0.200 |
| **Baseline H-score** | 1.00 (0.98 to 1.01) | 0.766 |  |  |
| **Underlying cause** |  |  |  |  |
| **Rheumatic disease** | 0.79 (0.23 to 2.79) | 0.718 |  |  |
| **Malignancy** | 0.41 (0.13 to 1.25) | 0.117 |  |  |
| **Infection** | 1.33 (0.41 to 4.30) | 0.637 |  |  |
| **Idiopathic** | 2.27 (0.73 to 7.01) | 0.156 |  |  |
| **Baseline fibrinogen** | 1.00 (1.00 to 1.00) | 0.040 | 1.00 (0.99 to 1.00) | 0.530 |
| **Treatment at baseline** |  |  |  |  |
| **HLH-2004 regimen** | 0.56 (0.20 to 1.53) | 0.258 |  |  |
| **Chemotherapy** | 0.81 (0.18 to 3.55) | 0.776 |  |  |
| **Other immunosuppressant** | 0.40 (0.06 to 2.24) | 0.284 |  |  |
| **Steroid only** | 2.79 (0.52 to 15.0) | 0.232 |  |  |
| **Tocilizumab** | 7.00 (0.81 to 60.7) | 0.077 | 12.93 (1.16 to 144.05) | 0.037 |

^*^, Adjusted for clinical factors with relevant association (P < 0.1) in the univariable analysis

GFR, glomerular filtration rate; MELD, Model For End-Stage Liver Disease; HLH, hemophagocytic lymphohistiocytosis

**Supplementary table S3. Risk factor for composite outcome at D14**

|  | **Univariable analysis** | | **Multivariable analysis** | |  |
| --- | --- | --- | --- | --- | --- |
|  | **OR (95% CI)** | **P** | **OR (95% CI)** | **P** |  |
| **Clinical factors** |  |  |  |  |  |
| **Age** | 1.05 (1.01 to 1.08) | **0.005** | 1.05 (1.01 to 1.09) | **0.026** |  |
| **Male sex** | 0.85 (0.31 to 2.32) | 0.749 |  |  |  |
| **Symptom duration** | 1.01 (0.99 to 1.03) | 0.17 |  |  |  |
| **Steroid prior to the treatment** | 0.97 (0.31 to 3.00) | 0.957 |  |  |  |
| **Baseline MELD** | 1.23 (1.12 to 1.36) | **<0.001** | 1.10 (10.99 to 1.21) | 0.064 |  |
| **Baseline GFR** | 0.97 (0.95 to 1.00) | 0.286 |  |  |  |
| **Baseline H-score** | 0.99 (0.97 to 1.00) | 0.132 |  |  |  |
| **Baseline fibrinogen** | 1.00 (0.99 to 1.00) | 0.126 |  |  |  |
| **Underlying cause** | |  |  |  |  |
| **Rheumatic disease** | 1.05 (0.30 to 3.77) | 0.935 |  |  |  |
| **Malignancy** | 0.20 (0.05 to 0.79) | **0.021** | 0.39 (0.06 to 2.35) | 0.303 |  |
| **Infection** | 0.97 (0.30 to 3.15) | 0.955 |  |  |  |
| **Idiopathic** | 3.80 (1.23 to 11.70) | **0.020** | 4.01 (0.83 to 19.43) | 0.085 |  |
| **Treatment at baseline** |  |  |  |  |  |
| **HLH-2004 regimen** | 0.76 (0.28 to 2.09) | 0.595 |  |  |  |
| **Chemotherapy** | 0.18 (0.02 to 1.54) | 0.116 |  |  |  |
| **Other immunosuppressant** | 0.26 (0.03 to 2.40) | 0.237 |  |  |  |
| **Steroid only** | 5.40 (1.00 to 29.30) | **0.051** | 3.76 (0.46 to 30.7) | 0.217 |  |
| **Tocilizumab** | 5.40 (1.00 to 29.30) | **0.051** | 16.35 (1.83 to 145.66) | **0.012** |  |

* Adjusted for clinical factors with relevant association (P < 0.1) in univariable analysis

GFR, glomerular filtration rate; HLH, hemophagocytic lymphohistiocytosis; MELD, Model For End-Stage Liver Disease

**Supplementary table S4 Risk factor for composite outcome at D28**

|  | **Univariable analysis** | | **Multivariable analysis** | |
| --- | --- | --- | --- | --- |
|  | **OR (95% CI)** | **P** | **OR (95% CI)** | **P** |
| **Clinical factors** |  |  |  |  |
| **Age** | 1.03 (1.00 to 1.06) | **0.036** | 1.03 (0.99 to 1.07) | 0.103 |
| **Male sex** | 0.54 (0.220to 1.46) | 0.536 |  |  |
| **Symptom duration** | 1.01 (0.99 to 1.03) | 0.358 |  |  |
| **Steroid prior to the treatment** | 0.78 (0.26 to 2.38) | 0.665 |  |  |
| **Baseline MELD** | 1.15 (1.05 to 1.26) | **0.002** | 1.20 (1.05 to 1.38) | **0.010** |
| **Baseline GFR** | 0.99 (0.98 to 1.00) | **0.038** | 1.00 (0.99 to 1.01) | 0.960 |
| **Baseline H-score** | 0.99 (0.98 to 1.01) | 0.389 |  |  |
| **Baseline fibrinogen** | 1.00 (0.99 to 1.00) | 0.152 |  |  |
| **Underlying cause** | |  |  |  |
| **Rheumatic disease** | 1.08 (0.31 to 3.79) | 0.904 |  |  |
| **Malignancy** | 0.30 (0.09 to 0.97) | **0.044** | 0.69 (0.14 to 3.47) | 0.649 |
| **Infection** | 0.91 (0.29 to 2.90) | 0.875 |  |  |
| **Idiopathic** | 3.25 (1.04 to 10.13) | **0.042** | 4.57 (0.85 to 24.48) | 0.076 |
| **Treatment at baseline** |  |  |  |  |
| **HLH-2004 regimen** | 0.61 (0.22 to 1.66) | 0.332 |  |  |
| **Chemotherapy** | 0.31 (0.06 to 1.67) | 0.173 |  |  |
| **Other immunosuppressant** | 0.50 (0.09 to 2.95) | 0.444 |  |  |
| **Steroid only** | 3.72 (0.69 to 20.06) | 0.126 |  |  |
| **Tocilizumab** | 9.33 (1.08 to 81.02) | **0.043** | 37.61 (2.81 to 503.66) | **0.006** |

* Adjusted for clinical factors with relevant association (P < 0.1) in univariable analysis

GFR, glomerular filtration rate; HLH, hemophagocytic lymphohistiocytosis; MELD, Model For End-Stage Liver Disease

**Supplementary table S5. Clinical factors significantly associated with 56-day overall survival in the subgroup of patients who were treated with tocilizumab or HLH-2004 regimen**

|  | **Univariable analysis** | | **Multivariable analysis** | |
| --- | --- | --- | --- | --- |
|  | **HR (95% CI)** | **P** | **HR (95% CI)** | **P** |
| **Clinical factors** |  |  |  |  |
| **Age** | 1.02 (1.00 to 1.05) | 0.117 |  |  |
| **Male sex** | 0.83 (0.37 to 1.88) | 0.655 |  |  |
| **Symptom duration** | 1.00 (0.99 to 1.01) | 0.836 |  |  |
| **Steroid use prior to the treatment** | 1.49 (0.44 to 5.02) | 0.521 |  |  |
| **Baseline MELD** | 1.08 (1.03 to 1.14) | **0.002** | 1.09 (1.02 to 1.18) | **0.018** |
| **Baseline GFR** | 0.99 (0.99 to 1.00) | **0.084** | 1.00 (0.99 to 1.01) | 0.738 |
| **Baseline H-score** | 1.00 (0.99 to 1.01) | 0.553 |  |  |
| **Baseline fibrinogen** | 0.99 (0.99 to 1.00) | **0.038** | 0.99 (0.99 to 1.00) | 0.117 |
| **Underlying cause** |  |  |  |  |
| **Rheumatic disease** | 0.93 (0.38 to 2.25) | 0.864 |  |  |
| **Malignancy** | 0.90 (0.33 to 2.41) | 0.827 |  |  |
| **Infection** | 0.65 (0.24 to 1.75) | 0.392 |  |  |
| **Idiopathic** | 1.58 (0.68 to 3.65) | 0.287 |  |  |
| **Treatment at baseline** |  |  |  |  |
| **Tocilizumab  (vs. HLH-2004 regimen)** | 3.10 (1.25 to 7.66) | **0.014** | 3.85 (1.40 to 10.61) | **0.009** |

* Adjusted for clinical factors with relevant association (P < 0.1) in univariable analysis

GFR, glomerular filtration rate; HLH, hemophagocytic lymphohistiocytosis; MELD, Model For End-Stage Liver Disease

**Supplementary figure S2. Kaplan-Meier curve comparing Day 56 overall survival in the subgroup of patients : tocilizumab versus HLH-2004 regimen**

**
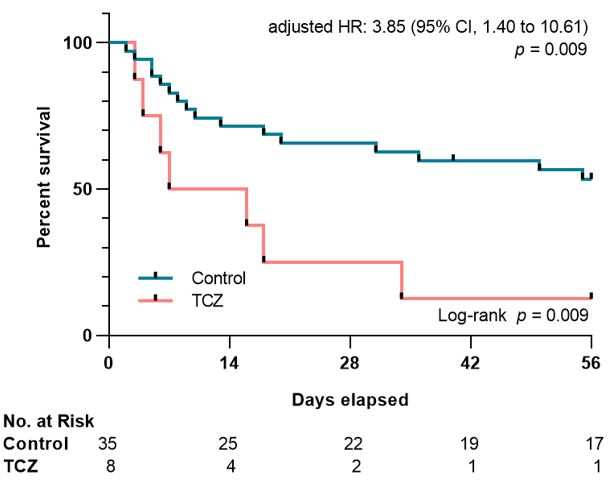
**

**Supplementary table S6. Clinical factors significantly associated with overall survival from the onset of initial HLH-related symptom**

|  | **Univariable analysis** | | **Multivariable analysis** | |
| --- | --- | --- | --- | --- |
|  | **HR (95% CI)** | **P** | **HR (95% CI)** | **P** |
| **Clinical factors** |  |  |  |  |
| **Age** | 1.03 (1.01 to 1.05) | **0.016** | 1.01 (0.99 to 1.04) | 0.250 |
| **Male sex** | 0.60 (0.30 to 1.18) | 0.139 |  |  |
| **Symptom duration** | 1.00 (0.99 to 1.01) | 0.843 |  |  |
| **Steroid use prior to the treatment** | 0.97 (0.45 to 2.09) | 0.939 |  |  |
| **Baseline MELD** | 1.07 (1.03 to 1.12) | **0.001** | 1.07 (1.01 to 1.13) | **0.021** |
| **Baseline GFR** | 0.99 (0.99 to 1.00) | **0.045** | 1.00 (0.99 to 1.01) | 0.682 |
| **Baseline H-score** | 1.00 (0.99 to 1.01) | 0.365 |  |  |
| **Baseline fibrinogen** | 1.00 (0.99 to 1.00) | 0.235 |  |  |
| **Underlying cause** |  |  |  |  |
| **Rheumatic disease** | 0.88 (0.36 to 2.13) | 0.778 |  |  |
| **Malignancy** | 0.72 (0.31 to 1.66) | 0.440 |  |  |
| **Infection** | 0.79 (0.34 to 1.82) | 0.580 |  |  |
| **Idiopathic** | 1.74 (0.87 to 3.51) | 0.120 |  |  |
| **Treatment at baseline** |  | |  |  |
| **HLH-2004 regimen** | 0.60 (0.30 to 1.19) | 0.146 |  |  |
| **Chemotherapy** | 0.63 (0.19 to 2.07) | 0.446 |  |  |
| **Other immunosuppressant** | 0.56 (0.13 to 2.35) | 0.428 |  |  |
| **Steroid only** | 2.38 (0.97 to 5.81) | **0.058** | 2.10 (0.79 to 5.59) | 0.139 |
| **Tocilizumab** | 3.51 (1.50 to 8.19) | **0.004** | 5.45 (2.11 to 14.08) | **<0.001** |

* Adjusted for clinical factors with relevant association (P < 0.1) in univariable analysis

GFR, glomerular filtration rate; HLH, hemophagocytic lymphohistiocytosis; MELD, Model For End-Stage Liver Disease

**Supplementary figure S3. Kaplan-Meier curve between the two groups from the onset of initial HLH-related symptom**


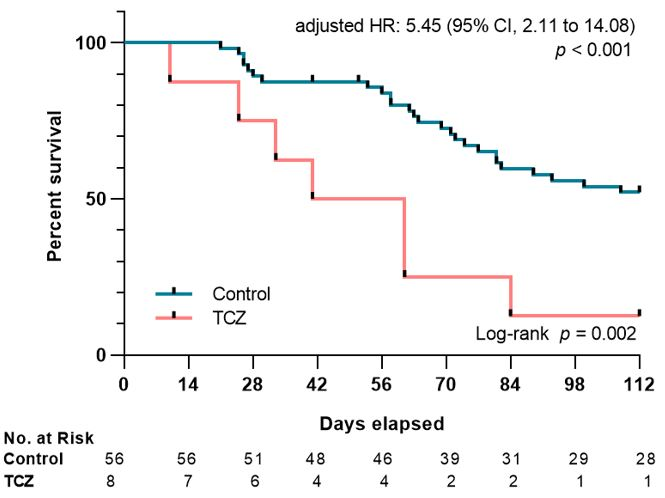


**Supplementary figure S4. HLH response based on a response criterion described in the emapalumab study**
